# 2,6-Di­chloro-9-(2′,3′,5′-tri-*O*-acetyl-β-d-ribo­furanos­yl)-9*H*-purine

**DOI:** 10.1107/S1600536813034521

**Published:** 2014-01-04

**Authors:** Irina Novosjolova, Dmitrijs Stepanovs, Ērika Bizdēna, Anatoly Mishnev, Māris Turks

**Affiliations:** a Department of Material Science and Applied Chemistry, Riga Technical University, 14/24 Azenes street, Riga, LV-1007, Latvia; b Latvian Institute of Organic Synthesis, 21 Aizkraukles street, Riga, LV-1006, Latvia

## Abstract

The title synthetic analog of purine nucleosides, C_16_H_16_Cl_2_N_4_O_7_, has its acetyl­ated β-furan­ose ring in a 3′β-envelope conformation, with the corresponding C atom deviating by 0.602 (5) Å from the rest of the ring. The planar part of the furan­ose ring forms a dihedral angle of 65.0 (1)° with the mean plane of the purine bicycle. In the crystal, mol­ecules form a three-dimensional network through multiple C—H⋯O and C—H⋯N hydrogen bonds and C—H⋯π interactions.

## Related literature   

For applications of 9-(2′,3′,5′-tri-*O*-acetyl-β-d-ribo­furanos­yl)-2,6-di­chloro-9*H*-purine in synthesis, see: Caner & Vilarrasa (2010 ▶); Korboukh *et al.* (2012 ▶). For the synthesis, see: Vorbrüggen (1995 ▶); Robins & Uznański (1981 ▶); Nair & Richardson (1982 ▶); Francom *et al.* (2002 ▶); Francom & Robins (2003 ▶); Gerster & Robins (1966 ▶). The conditions were improved by using our previous studies (Kovalovs *et al.*, 2013 ▶; Novosjolova *et al.*, 2013 ▶). For the biological activity of purine nucleosides, their anti­cancer and anti­viral activity and use as agonists and antagonists of adenosine receptors, see: Lech-Maranda *et al.* (2006 ▶); Robak *et al.* (2009 ▶); Gumina *et al.* (2003 ▶); Fredholm *et al.* (2011 ▶); Elzein & Zablocki (2008 ▶). For the structure of another 2,6-di­chloro­purine ribonucleoside, 9-(2′-de­oxy-3′,5′-di-*O*-4-meth­oxy­benzoyl-β-d-ribo­furanos­yl)-2,6-di­chloro-9*H*-purine, see:Yang *et al.* (2012 ▶). The purine heterocycle is known to form π–π stacking inter­actions in related structures, see: Sternglanz & Bugg (1975 ▶). For standard bond lengths, see: Allen *et al.* (1987 ▶). The nature of hydrogen bonding is described by Gilli (2002 ▶). For a description of the Cambridge Structural Database, see: Allen (2002 ▶).
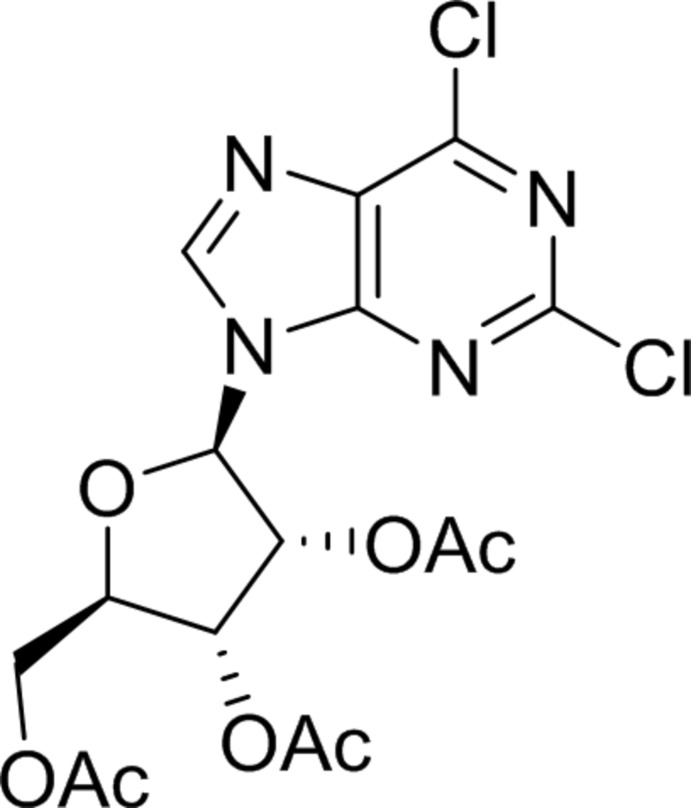


## Experimental   

### 

#### Crystal data  

C_16_H_16_Cl_2_N_4_O_7_
*M* *_r_* = 447.23
Monoclinic, 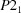

*a* = 10.1324 (2) Å
*b* = 9.6887 (3) Å
*c* = 10.5399 (2) Å
β = 106.537 (2)°
*V* = 991.90 (4) Å^3^
*Z* = 2
Mo *K*α radiation
μ = 0.37 mm^−1^
*T* = 296 K
0.38 × 0.32 × 0.15 mm

#### Data collection  

Nonius KappaCCD diffractometer
3898 measured reflections
3898 independent reflections
2846 reflections with *I* > 2σ(*I*)

#### Refinement  

*R*[*F* ^2^ > 2σ(*F* ^2^)] = 0.046
*wR*(*F* ^2^) = 0.107
*S* = 1.02
3898 reflections
265 parameters
H-atom parameters constrained
Δρ_max_ = 0.22 e Å^−3^
Δρ_min_ = −0.20 e Å^−3^
Absolute structure: Flack (1983 ▶), 1518 Friedel pairs
Absolute structure parameter: 0.00 (7)

### 

Data collection: *KappaCCD Server Software* (Nonius, 1997 ▶); cell refinement: *SCALEPACK* (Otwinovski & Minor, 1997 ▶); data reduction: *DENZO* (Otwinovski & Minor, 1997 ▶) and *SCALEPACK*; program(s) used to solve structure: *SHELXS97* (Sheldrick, 2008 ▶); program(s) used to refine structure: *SHELXL97* (Sheldrick, 2008 ▶); molecular graphics: *ORTEP-3 for Windows* (Farrugia, 2012 ▶) and *Mercury* (Macrae *et al.*, 2008 ▶); software used to prepare material for publication: *SHELXL97*, *PLATON* (Spek, 2009 ▶) and *publCIF* (Westrip, 2010 ▶).

## Supplementary Material

Crystal structure: contains datablock(s) I. DOI: 10.1107/S1600536813034521/ld2116sup1.cif

Structure factors: contains datablock(s) I. DOI: 10.1107/S1600536813034521/ld2116Isup2.hkl

CCDC reference:

Additional supporting information:  crystallographic information; 3D view; checkCIF report

## Figures and Tables

**Table 1:** Hydrogen-bond geometry (Å, °) *Cg* is the centroid of the C4/C5/N7/C8/N9 imidazole ring.

*D*—H⋯*A*	*D*—H	H⋯*A*	*D*⋯*A*	*D*—H⋯*A*
C8—H8⋯O15′	0.93	2.52	3.265 (4)	137
C8—H8⋯O14′^i^	0.93	2.56	3.350 (4)	143
C1′—H1⋯N1^ii^	0.98	2.48	3.355 (5)	148
C9′—H9*B*⋯O18′^iii^	0.96	2.51	3.434 (4)	161
C13′—H13*B*⋯N7^iv^	0.96	2.54	3.502 (5)	175
C5′—H5*A*⋯*Cg* ^i^	0.97	2.69	3.454	136

Symmetry codes: (i) 
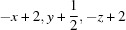
; (ii) 
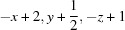
; (iii) 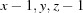; (iv) 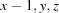.

**Fig. 1.**
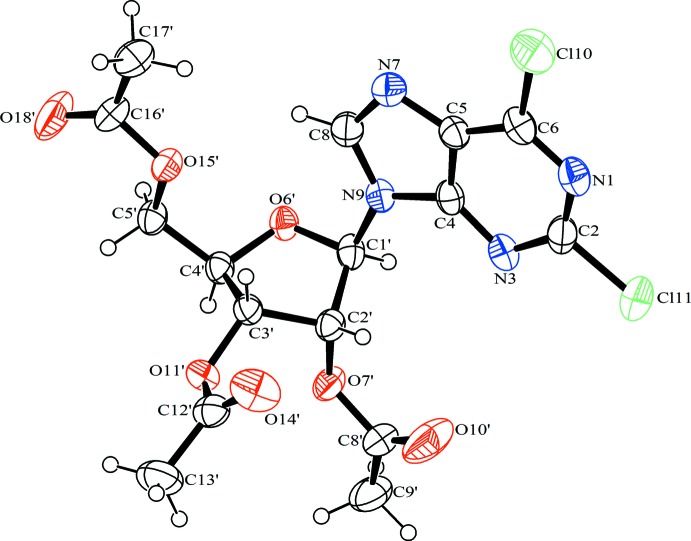
The asymmetric unit of the title compound showing 50% probability displacement ellipsoids and the atom-numbering (hydrogen atoms are shown as small spheres of arbitrary radii)

**Fig. 2.**
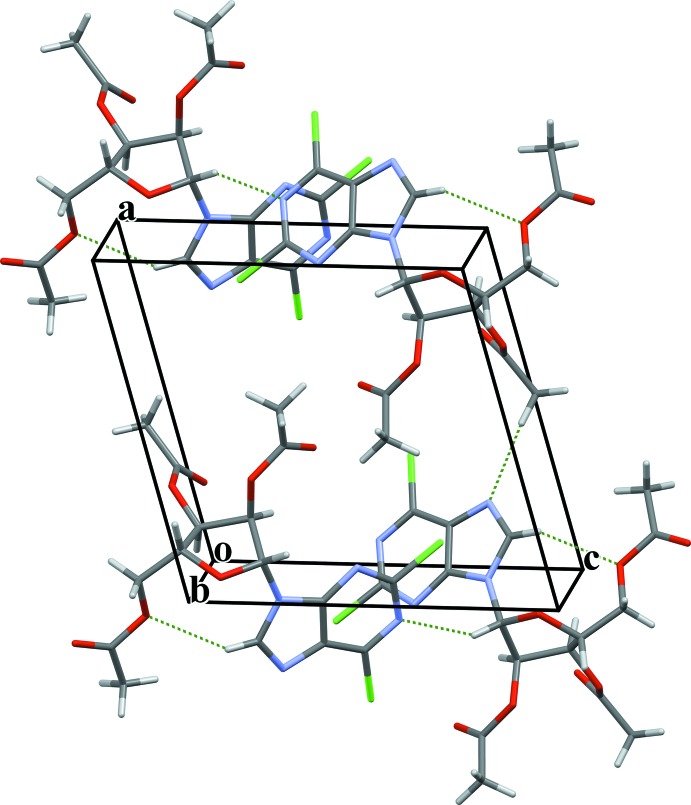
Packing diagram of the title compound viewed down the b axis

## supplementary crystallographic information

### 1. 1. Comment

Purine nucleosides are well known of their biological activity: anti­cancer and anti­viral activities, and as agonists and antagonists of adenosine receptors (Lech-Maranda *et al.* (2006), Robak *et al.* (2009), Gumina *et al.* (2003), Fredholm *et al.* (2011), Elzein & Zablocki (2008).

There is only one example of similar crystal structure of 2,6-di­chloro­purine ribonucleoside (9-(2'-de­oxy-3',5'-di-*O*-4-meth­oxy­benzoyl-β-*D*-ribo­furan­osyl)-2,6-di­chloro-9*H*-purine) in literature (Yang *et al.*, 2012, CSD refcode KEBWOF). Search of the Cambridge Structural Database (CSD, Version 1.15; Allen, 2002) indicated that there are only 4 (CSD refcodes: CLPURB, JEMHUF, PUPZAC, ZEXWEE) entries of 2- or 6-chloro substituted derivatives from over 300 crystal structures of 9-(ribo­furan­osyl)-9*H*-purines. The bond lengths (Allen *et al.*, 1987) and angles in the molecule of are close to standard values. The furan­ose cycle adopts an envelope conformation. Atoms C2', C1', O6' and C4' of furan­ose lie in same plane (denoted as plane *A*), 2'- and 5'-*O*-acetyl groups are on opposite sides of the *A* plane, while 3'-*O*-acetyl group lies close to the *A* plane. The C3' deviates from the *A* by 0.602 (5) Å. The dihedral angle between the least-square planes of the purine system and four planar atoms (C2', C1', O6' and C4') of furan­ose cycle is 65.0 (1)°. The main torsion angles describing the location of purine system in respect to furan­ose ring are: C8—N9—C1'—O6' (6.9 (4)°); C8—N9—C1'—C2' (-111.1 (3)°); C4—N9—C1'—O6' (-168.8 (3)°); C4—N9—C1'—C2' (73.2 (4)°). Regardless of the fact that purine heterocycle is known to form π–π stacking inter­actions in related structures (Sternglanz & Bugg, 1975), such inter­action was not observed in the crystals of the title compound. Moderate hydrogen bonds type C—H···O and C— H···N are present to form and stabilize three-dimensional architecture (Table 1).

The 9-(2',3',5'-tri-*O*-acetyl-β-*D*-ribo­furan­osyl)-2,6-di­chloro-9*H*-purine 1 was synthesized by method of Vorbrüggen glycosyl­ation of 2,6-di­chloro­purine (Vorbrüggen, 1995). The conditions were improved by using our previous studies (Kovalovs *et al.*, 2013; Novosjolova *et al*., 2013).

### 2. 2. Experimental

Single crystals of 9-(2',3',5'-tri-*O*-acetyl-β-*D*-ribo­furan­osyl)-2,6-di­chloro-9*H*-purine were grown from an ethanol solution by slow evaporation in ambient temperature. ^1^H-NMR and ^13^C-NMR spectra were recorded at 300 MHz and at 75.5 MHz, respectively. The proton signals for residual non-deuterated solvents (δ 7.26 for CDCl_3_) and carbon signals (δ 77.1 for CDCl_3_) were used as an inter­nal references for ^1^H-NMR and ^13^C-NMR spectra, respectively. Coupling constants are reported in Hz. Analytical thin layer chromatography (TLC) was performed on Kieselgel 60 F254 glass plates precoated with a 0.25 mm thickness of silica gel. Dry MeCN was obtained by distillation over CaH_2_. Commercial reagents were used as received.

9-(2',3',5'-Tri-*O*-acetyl-β-*D*-ribo­furan­osyl)-2,6-di­chloro-9*H*-purine (3). *N*,*O*-Bis(tri­methyl­silyl)acetamide (5.60 mL, 22.7 mmol) was added to a stirred suspension of 2,6-di­chloro­purine (2) (4.02 g, 21.2 mmol) in dry aceto­nitrile (50 mL). The resulting mixture was stirred at 40 °C for 30 min until a clear solution was obtained. Solution of tetra-*O*-acetyl-D-ribo­furan­ose (1) (6.77 g, 21.3 mmol) in dry aceto­nitrile (35 mL) was then added, followed by TMSOTf (0.80 mL, 4.4 mmol). The resulting reaction mixture was stirred at 75-80 °C for 2.5-3 h (TLC control). Then it was cooled to ambient temperature and ethanol (1 mL) was added and the mixture was stirred for 15 min at the same temperature followed by evaporation under reduced pressure. The residue was dissolved in CH_2_Cl_2_ (100 mL) washed with saturated aqueous solution of NaHCO_3_ (3 × 25 mL) and water (1 × 25 mL), dried over anh. Na_2_SO_4_. Evaporation under reduced pressure provided product 3 (8.95 g, 95%) as a slightly yellow powder. The title compound was crystallized from ethanol, mp 158-160 °C [lit.: 159-161 °C (Gerster & Robins, 1966)]. The other analytical data of 9-(2',3',5'-tri-*O*-acetyl-β-*D*-ribo­furan­osyl)-2,6-di­chloro-9*H*-purine are consistent with those reported earlier (Francom *et al.*, 2002; Caner & Vilarrasa, 2010). *R*_f_=0.45 (Toluene/EtOAc 1:2), IR (KBr), ν, cm^-1^: 2976, 2924, 1773, 1742, 1593, 1560, 1381, 1367, 1245, 1229, 1200, 1137, 1100, 1061; ^1^H-NMR (300 MHz, CDCl_3_) δ (ppm): 8.29 (s, 1H, H—C(8)), 6.21 (d, 1H, ^3^*J*_1'-2'_ = 5.6 Hz, H—C(1')), 5.79 (t, 1H, ^3^*J*_1'-2'_ = ^3^*J*_2'-3'_ = 5.6 Hz, H—C(2')), 5.57 (dd, 1H, ^3^*J*_2'-3'_ = 5.6 Hz, ^3^*J*_3'-4'_ = 4.4 Hz, H—C(3')), 4.47 (quartet, 1H, ^3^*J*_3'-4'_ = ^3^*J*_4'-5'_ = 4.4 Hz, H—C(4')), 4.41 (d, 2H, ^3^*J*_4'-5'_ = 4.4 Hz, H—C(5')), 2.16, 2.14, 2.09 (3s, 9H, H_3_COOC-C(2',3',5')); ^13^C-NMR (75.5 MHz, CDCl_3_) δ (ppm): 170.2, 169.5, 169.4, 153.3, 152.6, 152.2, 143.9, 131.3, 86.5, 80.8, 73.2, 70.5, 62.8, 20.7, 20.5, 20.3.

### 2.1. 3. Refinement

Crystal data, data collection and structure refinement details are summarized in Table 1. All non-hydrogen atoms were refined anisotropically. All hydrogen atoms were positioned geometrically with C—H distances ranging from 0.93 Å to 0.98 Å and refined as riding on their parent atoms with *U*_iso_ (H) = 1.5*U*_eq_ (C) for methyl groups and *U*_iso_ (H) = 1.2*U*_eq_ (C) for others.

### Crystal data

**Table:**

C_16_H_16_Cl_2_N_4_O_7_	*F*(000) = 460
*M**_r_* = 447.23	*D*_x_ = 1.497 Mg m^−^^3^
Monoclinic, *P*2_1_	Mo *K*α radiation, λ = 0.71073 Å
Hall symbol: P 2yb	Cell parameters from 17407 reflections
*a* = 10.1324 (2) Å	θ = 1.0–27.5°
*b* = 9.6887 (3) Å	µ = 0.37 mm^−^^1^
*c* = 10.5399 (2) Å	*T* = 296 K
β = 106.537 (2)°	Prism, colorless
*V* = 991.90 (4) Å^3^	0.38 × 0.32 × 0.15 mm
*Z* = 2	

### Data collection

**Table:**

Nonius KappaCCD diffractometer	2846 reflections with *I* > 2σ(*I*)
Radiation source: fine-focus sealed tube	*R*_int_ = 0.000
Graphite monochromator	θ_max_ = 27.5°, θ_min_ = 2.0°
CCD scans	*h* = −13→13
3898 measured reflections	*k* = −11→12
3898 independent reflections	*l* = −13→13

### Refinement

**Table:**

Refinement on *F*^2^	Secondary atom site location: difference Fourier map
Least-squares matrix: full	Hydrogen site location: inferred from neighbouring sites
*R*[*F*^2^ > 2σ(*F*^2^)] = 0.046	H-atom parameters constrained
*wR*(*F*^2^) = 0.107	*w* = 1/[σ^2^(*F*_o_^2^) + (0.0352*P*)^2^ + 0.3514*P*] where *P* = (*F*_o_^2^ + 2*F*_c_^2^)/3
*S* = 1.02	(Δ/σ)_max_ < 0.001
3898 reflections	Δρ_max_ = 0.22 e Å^−^^3^
265 parameters	Δρ_min_ = −0.20 e Å^−^^3^
0 restraints	Absolute structure: Flack (1983), 1518 Friedel pairs
0 constraints	Absolute structure parameter: 0.00 (7)
Primary atom site location: structure-invariant direct methods	

### Special details

**Table:**

Geometry. All e.s.d.'s (except the e.s.d. in the dihedral angle between two l.s. planes) are estimated using the full covariance matrix. The cell e.s.d.'s are taken into account individually in the estimation of e.s.d.'s in distances, angles and torsion angles; correlations between e.s.d.'s in cell parameters are only used when they are defined by crystal symmetry. An approximate (isotropic) treatment of cell e.s.d.'s is used for estimating e.s.d.'s involving l.s. planes.
Refinement. Refinement of *F*^2^ against ALL reflections. The weighted *R*-factor *wR* and goodness of fit *S* are based on *F*^2^, conventional *R*-factors *R* are based on *F*, with *F* set to zero for negative *F*^2^. The threshold expression of *F*^2^ > σ(*F*^2^) is used only for calculating *R*-factors(gt) *etc*. and is not relevant to the choice of reflections for refinement. *R*-factors based on *F*^2^ are statistically about twice as large as those based on *F*, and *R*- factors based on ALL data will be even larger.

### Fractional atomic coordinates and isotropic or equivalent isotropic displacement parameters (Å^2^)

**Table:**

	*x*	*y*	*z*	*U*_iso_*/*U*_eq_	
N1	1.0850 (3)	0.1139 (3)	0.4785 (3)	0.0535 (8)	
C2	0.9650 (4)	0.1787 (4)	0.4602 (3)	0.0508 (9)	
N3	0.9178 (3)	0.2553 (3)	0.5399 (2)	0.0458 (6)	
C4	1.0170 (3)	0.2704 (3)	0.6574 (3)	0.0404 (7)	
C5	1.1477 (3)	0.2135 (4)	0.6915 (3)	0.0429 (8)	
C6	1.1761 (4)	0.1309 (4)	0.5952 (3)	0.0510 (9)	
N7	1.2211 (3)	0.2508 (3)	0.8196 (3)	0.0506 (7)	
C8	1.1331 (3)	0.3266 (4)	0.8596 (3)	0.0473 (8)	
H8	1.1540	0.3671	0.9430	0.057*	
N9	1.0071 (2)	0.3404 (3)	0.7669 (2)	0.0405 (6)	
Cl10	1.32958 (12)	0.04548 (15)	0.62312 (11)	0.0892 (4)	
Cl11	0.85038 (11)	0.15549 (12)	0.30379 (9)	0.0733 (3)	
C1'	0.8877 (3)	0.4205 (4)	0.7769 (3)	0.0409 (7)	
H1	0.8574	0.4827	0.7008	0.049*	
C2'	0.7695 (3)	0.3282 (4)	0.7845 (3)	0.0397 (7)	
H2	0.7642	0.2421	0.7344	0.048*	
C3'	0.8034 (3)	0.3062 (3)	0.9343 (3)	0.0385 (7)	
H3	0.8772	0.2380	0.9639	0.046*	
C4'	0.8537 (3)	0.4472 (3)	0.9862 (3)	0.0382 (7)	
H4	0.7733	0.5063	0.9786	0.046*	
C5'	0.9455 (3)	0.4570 (4)	1.1251 (3)	0.0432 (8)	
H5A	0.9788	0.5509	1.1437	0.052*	
H5B	0.8946	0.4325	1.1870	0.052*	
O6'	0.9264 (2)	0.4982 (2)	0.89486 (18)	0.0402 (5)	
O7'	0.6460 (2)	0.4081 (2)	0.74576 (19)	0.0490 (6)	
C8'	0.5479 (3)	0.3728 (4)	0.6337 (3)	0.0520 (9)	
C9'	0.4297 (4)	0.4704 (5)	0.6096 (4)	0.0696 (12)	
H9A	0.4616	0.5627	0.6031	0.104*	
H9C	0.3895	0.4651	0.6816	0.104*	
H9B	0.3620	0.4462	0.5285	0.104*	
O10'	0.5636 (3)	0.2809 (4)	0.5649 (3)	0.0967 (11)	
O11'	0.6883 (2)	0.2701 (2)	0.9804 (2)	0.0458 (6)	
C12'	0.6474 (4)	0.1370 (4)	0.9636 (3)	0.0504 (9)	
C13'	0.5286 (4)	0.1098 (5)	1.0189 (5)	0.0826 (15)	
H13A	0.5188	0.0122	1.0288	0.124*	
H13B	0.4456	0.1462	0.9597	0.124*	
H13C	0.5455	0.1538	1.1037	0.124*	
O14'	0.7012 (3)	0.0544 (3)	0.9110 (3)	0.0775 (8)	
O15'	1.0599 (2)	0.3644 (2)	1.13994 (18)	0.0441 (5)	
C16'	1.1551 (3)	0.3677 (4)	1.2595 (3)	0.0499 (8)	
C17'	1.2746 (4)	0.2794 (4)	1.2628 (3)	0.0618 (10)	
H17A	1.3570	0.3342	1.2865	0.093*	
H17B	1.2630	0.2393	1.1770	0.093*	
H17C	1.2816	0.2075	1.3270	0.093*	
O18'	1.1385 (3)	0.4370 (4)	1.3478 (2)	0.0909 (10)	

### Atomic displacement parameters (Å^2^)

**Table:**

	*U*^11^	*U*^22^	*U*^33^	*U*^12^	*U*^13^	*U*^23^
N1	0.063 (2)	0.055 (2)	0.0498 (17)	0.0015 (15)	0.0270 (14)	−0.0050 (14)
C2	0.064 (2)	0.052 (2)	0.0376 (17)	−0.0083 (19)	0.0173 (14)	−0.0028 (16)
N3	0.0551 (17)	0.0437 (17)	0.0444 (15)	0.0010 (13)	0.0239 (12)	0.0023 (13)
C4	0.0511 (19)	0.0378 (19)	0.0362 (16)	−0.0014 (15)	0.0189 (13)	0.0051 (14)
C5	0.0451 (19)	0.046 (2)	0.0406 (16)	0.0016 (15)	0.0166 (13)	0.0040 (15)
C6	0.057 (2)	0.050 (2)	0.0533 (19)	0.0053 (17)	0.0275 (16)	0.0000 (17)
N7	0.0465 (16)	0.0552 (19)	0.0495 (15)	0.0014 (14)	0.0126 (12)	−0.0009 (14)
C8	0.050 (2)	0.048 (2)	0.0437 (17)	−0.0015 (16)	0.0143 (14)	−0.0012 (15)
N9	0.0450 (15)	0.0425 (16)	0.0363 (13)	0.0033 (12)	0.0154 (11)	0.0016 (12)
Cl10	0.0712 (7)	0.1099 (11)	0.0907 (7)	0.0324 (7)	0.0299 (6)	−0.0156 (7)
Cl11	0.0824 (7)	0.0903 (9)	0.0433 (5)	−0.0046 (6)	0.0118 (4)	−0.0139 (5)
C1'	0.0501 (18)	0.0420 (19)	0.0319 (14)	0.0100 (15)	0.0137 (12)	0.0040 (14)
C2'	0.0413 (17)	0.0406 (19)	0.0364 (15)	0.0043 (14)	0.0096 (12)	−0.0049 (14)
C3'	0.0383 (16)	0.0419 (19)	0.0366 (15)	0.0032 (14)	0.0130 (12)	−0.0013 (14)
C4'	0.0405 (16)	0.0397 (19)	0.0357 (14)	0.0054 (14)	0.0129 (12)	−0.0043 (14)
C5'	0.0492 (18)	0.044 (2)	0.0364 (15)	0.0030 (15)	0.0116 (13)	−0.0055 (14)
O6'	0.0493 (12)	0.0367 (13)	0.0371 (11)	−0.0013 (10)	0.0162 (9)	−0.0035 (9)
O7'	0.0426 (12)	0.0597 (17)	0.0380 (11)	0.0133 (11)	0.0005 (9)	−0.0104 (10)
C8'	0.044 (2)	0.060 (3)	0.0451 (18)	−0.0044 (17)	0.0028 (14)	−0.0038 (18)
C9'	0.047 (2)	0.084 (3)	0.065 (2)	0.011 (2)	−0.0044 (16)	−0.006 (2)
O10'	0.079 (2)	0.096 (3)	0.089 (2)	0.0098 (18)	−0.0174 (16)	−0.048 (2)
O11'	0.0382 (12)	0.0531 (16)	0.0492 (12)	−0.0009 (11)	0.0174 (9)	0.0004 (11)
C12'	0.044 (2)	0.054 (2)	0.0484 (18)	−0.0001 (18)	0.0050 (14)	0.0148 (18)
C13'	0.053 (3)	0.109 (4)	0.087 (3)	−0.016 (2)	0.022 (2)	0.027 (3)
O14'	0.0762 (19)	0.0504 (18)	0.112 (2)	−0.0012 (16)	0.0364 (17)	0.0004 (17)
O15'	0.0455 (12)	0.0469 (14)	0.0356 (10)	0.0033 (10)	0.0049 (9)	−0.0040 (10)
C16'	0.051 (2)	0.048 (2)	0.0401 (18)	−0.0036 (17)	−0.0029 (14)	−0.0044 (17)
C17'	0.056 (2)	0.062 (3)	0.057 (2)	0.006 (2)	0.0006 (16)	0.0030 (19)
O18'	0.087 (2)	0.120 (3)	0.0482 (14)	0.026 (2)	−0.0101 (13)	−0.0346 (18)

### Geometric parameters (Å, º)

**Table:**

N1—C6	1.321 (4)	C4'—C5'	1.497 (4)
N1—C2	1.333 (4)	C4'—H4	0.9800
C2—N3	1.308 (4)	C5'—O15'	1.439 (4)
C2—Cl11	1.740 (3)	C5'—H5A	0.9700
N3—C4	1.363 (4)	C5'—H5B	0.9700
C4—N9	1.367 (4)	O7'—C8'	1.354 (4)
C4—C5	1.384 (4)	C8'—O10'	1.187 (4)
C5—C6	1.386 (4)	C8'—C9'	1.489 (5)
C5—N7	1.391 (4)	C9'—H9A	0.9600
C6—Cl10	1.712 (4)	C9'—H9C	0.9600
N7—C8	1.314 (4)	C9'—H9B	0.9600
C8—N9	1.376 (4)	O11'—C12'	1.351 (5)
C8—H8	0.9300	C12'—O14'	1.190 (4)
N9—C1'	1.466 (4)	C12'—C13'	1.502 (5)
C1'—O6'	1.409 (4)	C13'—H13A	0.9600
C1'—C2'	1.515 (5)	C13'—H13B	0.9600
C1'—H1	0.9800	C13'—H13C	0.9600
C2'—O7'	1.429 (4)	O15'—C16'	1.352 (3)
C2'—C3'	1.532 (4)	C16'—O18'	1.198 (4)
C2'—H2	0.9800	C16'—C17'	1.475 (5)
C3'—O11'	1.429 (3)	C17'—H17A	0.9600
C3'—C4'	1.505 (5)	C17'—H17B	0.9600
C3'—H3	0.9800	C17'—H17C	0.9600
C4'—O6'	1.455 (3)		
			
C6—N1—C2	116.3 (3)	C5'—C4'—C3'	117.7 (3)
N3—C2—N1	131.2 (3)	O6'—C4'—H4	108.3
N3—C2—Cl11	114.5 (3)	C5'—C4'—H4	108.3
N1—C2—Cl11	114.3 (2)	C3'—C4'—H4	108.3
C2—N3—C4	109.5 (3)	O15'—C5'—C4'	108.9 (2)
N3—C4—N9	127.5 (3)	O15'—C5'—H5A	109.9
N3—C4—C5	126.6 (3)	C4'—C5'—H5A	109.9
N9—C4—C5	105.9 (3)	O15'—C5'—H5B	109.9
C4—C5—C6	115.0 (3)	C4'—C5'—H5B	109.9
C4—C5—N7	110.9 (3)	H5A—C5'—H5B	108.3
C6—C5—N7	134.1 (3)	C1'—O6'—C4'	109.7 (2)
N1—C6—C5	121.2 (3)	C8'—O7'—C2'	118.5 (3)
N1—C6—Cl10	117.4 (2)	O10'—C8'—O7'	122.0 (3)
C5—C6—Cl10	121.4 (3)	O10'—C8'—C9'	127.8 (3)
C8—N7—C5	103.5 (3)	O7'—C8'—C9'	110.1 (3)
N7—C8—N9	113.8 (3)	C8'—C9'—H9A	109.5
N7—C8—H8	123.1	C8'—C9'—H9C	109.5
N9—C8—H8	123.1	H9A—C9'—H9C	109.5
C4—N9—C8	105.9 (3)	C8'—C9'—H9B	109.5
C4—N9—C1'	125.7 (2)	H9A—C9'—H9B	109.5
C8—N9—C1'	128.2 (3)	H9C—C9'—H9B	109.5
O6'—C1'—N9	108.5 (2)	C12'—O11'—C3'	116.1 (3)
O6'—C1'—C2'	107.2 (2)	O14'—C12'—O11'	122.6 (3)
N9—C1'—C2'	111.8 (3)	O14'—C12'—C13'	126.0 (4)
O6'—C1'—H1	109.8	O11'—C12'—C13'	111.5 (4)
N9—C1'—H1	109.8	C12'—C13'—H13A	109.5
C2'—C1'—H1	109.8	C12'—C13'—H13B	109.5
O7'—C2'—C1'	107.8 (3)	H13A—C13'—H13B	109.5
O7'—C2'—C3'	106.9 (2)	C12'—C13'—H13C	109.5
C1'—C2'—C3'	100.8 (2)	H13A—C13'—H13C	109.5
O7'—C2'—H2	113.5	H13B—C13'—H13C	109.5
C1'—C2'—H2	113.5	C16'—O15'—C5'	115.2 (2)
C3'—C2'—H2	113.5	O18'—C16'—O15'	121.1 (3)
O11'—C3'—C4'	108.8 (2)	O18'—C16'—C17'	127.1 (3)
O11'—C3'—C2'	114.8 (2)	O15'—C16'—C17'	111.8 (3)
C4'—C3'—C2'	101.7 (2)	C16'—C17'—H17A	109.5
O11'—C3'—H3	110.4	C16'—C17'—H17B	109.5
C4'—C3'—H3	110.4	H17A—C17'—H17B	109.5
C2'—C3'—H3	110.4	C16'—C17'—H17C	109.5
O6'—C4'—C5'	109.5 (2)	H17A—C17'—H17C	109.5
O6'—C4'—C3'	104.5 (2)	H17B—C17'—H17C	109.5
			
C6—N1—C2—N3	−2.3 (6)	O6'—C1'—C2'—O7'	82.0 (3)
C6—N1—C2—Cl11	178.7 (3)	N9—C1'—C2'—O7'	−159.2 (2)
N1—C2—N3—C4	3.0 (5)	O6'—C1'—C2'—C3'	−29.8 (3)
Cl11—C2—N3—C4	−178.0 (2)	N9—C1'—C2'—C3'	89.0 (3)
C2—N3—C4—N9	−178.6 (3)	O7'—C2'—C3'—O11'	44.0 (4)
C2—N3—C4—C5	−0.9 (5)	C1'—C2'—C3'—O11'	156.5 (3)
N3—C4—C5—C6	−1.5 (5)	O7'—C2'—C3'—C4'	−73.3 (3)
N9—C4—C5—C6	176.6 (3)	C1'—C2'—C3'—C4'	39.2 (3)
N3—C4—C5—N7	180.0 (3)	O11'—C3'—C4'—O6'	−157.0 (2)
N9—C4—C5—N7	−1.9 (4)	C2'—C3'—C4'—O6'	−35.5 (3)
C2—N1—C6—C5	−0.8 (5)	O11'—C3'—C4'—C5'	81.3 (3)
C2—N1—C6—Cl10	178.4 (3)	C2'—C3'—C4'—C5'	−157.2 (2)
C4—C5—C6—N1	2.4 (5)	O6'—C4'—C5'—O15'	−65.6 (3)
N7—C5—C6—N1	−179.6 (4)	C3'—C4'—C5'—O15'	53.4 (3)
C4—C5—C6—Cl10	−176.8 (3)	N9—C1'—O6'—C4'	−112.7 (3)
N7—C5—C6—Cl10	1.2 (6)	C2'—C1'—O6'—C4'	8.2 (3)
C4—C5—N7—C8	0.9 (4)	C5'—C4'—O6'—C1'	144.5 (2)
C6—C5—N7—C8	−177.2 (4)	C3'—C4'—O6'—C1'	17.6 (3)
C5—N7—C8—N9	0.4 (4)	C1'—C2'—O7'—C8'	115.0 (3)
N3—C4—N9—C8	−179.9 (3)	C3'—C2'—O7'—C8'	−137.4 (3)
C5—C4—N9—C8	2.0 (3)	C2'—O7'—C8'—O10'	−2.6 (5)
N3—C4—N9—C1'	−3.4 (5)	C2'—O7'—C8'—C9'	−178.7 (3)
C5—C4—N9—C1'	178.5 (3)	C4'—C3'—O11'—C12'	−168.0 (2)
N7—C8—N9—C4	−1.6 (4)	C2'—C3'—O11'—C12'	78.9 (3)
N7—C8—N9—C1'	−177.9 (3)	C3'—O11'—C12'—O14'	−1.6 (5)
C4—N9—C1'—O6'	−168.8 (3)	C3'—O11'—C12'—C13'	178.4 (3)
C8—N9—C1'—O6'	6.9 (4)	C4'—C5'—O15'—C16'	176.9 (3)
C4—N9—C1'—C2'	73.2 (4)	C5'—O15'—C16'—O18'	4.7 (5)
C8—N9—C1'—C2'	−111.1 (3)	C5'—O15'—C16'—C17'	−174.8 (3)

### Hydrogen-bond geometry (Å, º)

Cg is the centroid of the C4/C5/N7/C8/N9 imidazole ring.

**Table:**

*D*—H···*A*	*D*—H	H···*A*	*D*···*A*	*D*—H···*A*
C8—H8···O15′	0.93	2.52	3.265 (4)	137
C8—H8···O14′^i^	0.93	2.56	3.350 (4)	143
C1′—H1···N1^ii^	0.98	2.48	3.355 (5)	148
C9′—H9*B*···O18′^iii^	0.96	2.51	3.434 (4)	161
C13′—H13*B*···N7^iv^	0.96	2.54	3.502 (5)	175
C5′—H5*A*···*Cg*^i^	0.97	2.69	3.454	136

Symmetry codes: (i) −*x*+2, *y*+1/2, −*z*+2; (ii) −*x*+2, *y*+1/2, −*z*+1; (iii) *x*−1, *y*, *z*−1; (iv) *x*−1, *y*, *z*.
